# Enhancing In Vitro Production of the Tree Fern *Cyathea delgadii* and Modifying Secondary Metabolite Profiles by LED Lighting

**DOI:** 10.3390/cells11030486

**Published:** 2022-01-30

**Authors:** Wojciech Tomaszewicz, Monika Cioć, Katarzyna Dos Santos Szewczyk, Małgorzata Grzyb, Wioleta Pietrzak, Bożena Pawłowska, Anna Mikuła

**Affiliations:** 1 Center for Biological Diversity Conservation in Powsin—Polish Academy of Sciences Botanical Garden, Prawdziwka 2, 02-973 Warsaw, Poland; m.grzyb@obpan.pl (M.G.); a.mikula@obpan.pl (A.M.); 2 Department of Ornamental Plants and Garden Art, University of Agriculture in Krakow, 29 Listopada 54, 31-425 Kraków, Poland; monika.cioc@urk.edu.pl (M.C.); bozena.pawlowska@urk.edu.pl (B.P.); 3 Department of Pharmaceutical Botany, Medical University of Lublin, Chodźki 1, 20-093 Lublin, Poland; k.szewczyk@umlub.pl (K.D.S.S.); wioletapietrzak@umlub.pl (W.P.)

**Keywords:** light-emitting diode, somatic embryogenesis, photosynthetic pigments, phenolic acids, antioxidant activity, tree fern

## Abstract

The tree ferns are an important component of tropical forests. In view of this, the enhancement of in vitro production of these plants is needed. Thus, the effect of different light-emitting diodes (LEDs) as well as control fluorescent lamps (Fl) and a 3-week-long period of darkness at the beginning of in vitro culture on micropropagation of the tree fern *Cyathea delgadii* Sternb. was analysed. Moreover, the photosynthetic pigment content and secondary metabolite profiles were estimated. The period of darkness contributed to a high production of somatic embryo-derived sporophytes and a low production of gametophytes. The formation of new sporophytes was stimulated by RBY (35% red, 15% blue, and 50% yellow) and B (100% blue) lights when the stipe explants or whole young sporophytes were used in the culture, respectively. The elongation of the roots and leaves was stimulated by RBfR light (35% red, 15% blue, and 50% far red), while root production increased under RBY light. The RB (70% red and 30% blue) and B lights stimulated the accumulation of chlorophyll better than Fl light. The most abundant metabolite found in the plant extracts was *trans*-5-*O*-caffeoylquinic acid (1.013 µg/mg of dry weight). The extract obtained from plants growing in a greenhouse had the best antioxidant activity.

## 1. Introduction

Light is an energy source for photosynthesis and also acts as an environmental factor in photomorphogenesis, affecting growth and development of plants [1]. Light is perceived by photoreceptors, including cryptochromes and phototropins, responding to blue and UV-A light and phytochromes, which react with red and far-red light [2]. Fluorescent lamps are conventionally used in plant tissue culture laboratories as a light source; however, they emit a redundant wide range of wavelengths [3,4]. Light-emitting diodes (LEDs) are an alternative to them in in vitro culturing. They are characterised by controlled narrow-spectrum illumination, which can provide only wavelengths that match to plant photoreceptors. LEDs also have a longer lifespan and lower heat emission in comparison to fluorescent lamps [3,5,6]. Many studies on seed plants have demonstrated the influence of different light spectra generated by LEDs on shoot and root formation, leaf anatomy, somatic embryo induction, chlorophyll content, and accumulation of secondary metabolites [7,8,9,10]. 

The tree ferns represent an important component of tropical forests [11,12]. Due to habitat loss and overexploitation, this splendid and relic group is surging towards rarity in their natural habitat, and for this reason they were placed under the appendix II of CITES (Convention of International Trade in Endangered Species of Wild Fauna and Flora) [13]. Tree ferns play an important role in human life due to their ornamental, food, and medical applications [14,15,16]. It has been shown that the extracts from the *Cyathea* genus plants have such properties as antimicrobial [17], larvicidal [18], phytotoxic [19], hepatoprotective [20], and antioxidant [21]. Thus, to fulfil the demand for the tree ferns, alternative ways for their propagation are increasingly being developed. According to our knowledge, 27 tree fern species have been introduced to in vitro cultures. The majority of them are represented by ferns from the *Cyathea* genus: *C. atrovirens*, *C. australis*, *C. brownie*, *C. capensis*, *C. contaminans*, *C, cooperi*, *C. corcovadensis*, *C. cunninghamii*, *C. dealbata*, *C. delgadii*, *C. dregei*, *C. gigantea*, *C. leichhardtiana*, *C. lepifera*, *C. phalerata*, *C. robertsiana*, *C. schanschin*, *C. smithii*, and *C. spinulosa* [22,23,24,25,26,27]. The in vitro cultures were also established for *Dicksonia antarctica*, *D. fibrosa*, *D. sellowiana*, *Cibotium glaucum*, *Ci. schiedei*, *Ci. barometz*, *Blechnum brasiliense,* and *Alsophila odonelliana* [27,28,29]. Tree ferns were multiplied in vitro, e.g., by zygotic embryogenesis [24], somatic embryogenesis (SE) [30], apogamy [31], organogenesis [32] and through the formation of green globular bodies [29]. Among these methods, SE is still poorly understood, although it seems to be a very efficient way to obtain ferns in in vitro cultures.

The production of tree fern *C. delgadii* via SE can be affected by several factors, such as explant type, composition of culture medium, photoperiod, and stress treatment [33,34]. The etiolation of donor plants is the most important factor influencing fern SE, since nonetiolated plants are not able to produce somatic embryos [33]. Etiolation changes the endogenous hormone and carbohydrate contents leading to the acquisition of embryogenic competence of epidermal cells of stipe explants [35]. Moreover, it was shown that light stimulates the production of gametophytes and has an impact on the development of juvenile plants [33]. Previous studies on the effect of light on ferns have focused mainly on spore germination [22,36], gametophyte development [36,37], phototropism [38], chloroplast relocation [39], and stomata opening [40], while knowledge about its impact on micropropagation and the accumulation of secondary metabolites in ferns is still lacking.

In the present study, we examined the influence of different LED light spectra, fluorescent lamps, and darkness on the sporophyte and gametophyte production and development in *C. delgadii*. Moreover, we analysed the photosynthetic pigment and secondary metabolite profiles and antioxidant activity of extracts prepared from plants growing under different light conditions.

## 2. Materials and Methods

### 2.1. Plant Material and Culture Conditions

The etiolated 5-month-old sporophytes of *C. delgadii* with 3–4 developed leaves were used as the source of plant material. They were cultured on hormone-free half-strength macro- and micronutrients Murashige and Skoog’s medium (1/2 MS) [41] supplemented with full composition of vitamins and 2% (*w*/*v*) sucrose. The medium was solidified with 0.7% plant agar. Its pH was adjusted to 5.8 before autoclaving. Fifty mL of the medium was poured into glass jars of 190 mL each. The cultures of donor sporophytes as well as experimental cultures were maintained in a climatic chamber at 24 ± 1 °C.

In the first experiment, the stipe explants measuring 2.5 mm in length, excised from the youngest leaves of etiolated sporophytes, were used for the culture initiation [34]. They were cultured on 1/2 MS medium supplemented with 1% (*w*/*v*) sucrose: (1) for 8 weeks under 16/8 h light/dark (8L) at different light qualities (as listed below) or (2) for the first 3 weeks under 24 h dark and then for the next 5 weeks under 16/8 h light/dark (3D/5L, light qualities listed below). Three Petri dishes, each with 20 explants, were cultured per light treatment.

The plant material was treated with nine light quality combinations:

(1) Fl—fluorescent lamps (Philips TL-D 36W/54)
(2) B—100% blue LED light (430 nm)
(3) R—100% red LED light (670 nm)
(4) RB—combination of red and blue LED lights (70%/30%)
(5) RBfR—combination of red, blue, and far-red (730 nm) LED lights (35%/15%/50%)
(6) RBY—combination of red, blue, and yellow (600 nm) LED lights (35%/15%/50%)
(7) RBUV—combination of red, blue, and UV (400 nm) LED lights (35%/15%/50%)
(8) RBG—combination of red, blue, and green (528 nm) LED lights (35%/15%/50%)
(9) Wh—white LED (1:1:1 2700 K:4500 K:5700 K)

Specially designed and constructed LED panels were used for the experiments [9]. Lighting parameters were set by digital DMX-512 protocol. The light intensity and quality were adjusted using an LI-250A light meter with a Q 50,604 sensor (LI-COR, Lincoln, NE, USA) and a BTS256 spectrometer (Gigahertz-Optik, Türkenfeld, Germany). The photosynthetic photon flux density (PPFD) was set at 40 μmol m^−2^ s^−1^ in all treatments (excluding the period of darkness).

In the second experiment, whole etiolated 5-month-old sporophytes of *C. delgadii* with 3–4 developed leaves were placed in 500 mL glass jars with 80 mL of 1/2 MS supplemented with 2% (*w*/*v*) sucrose. They were cultured for 6 months under 16/8 h light/dark using 4 light combinations: Fl, B, R, and RB. The regenerative ability and morphology of plants were characterised after 6 months of the culture. Six jars, each with four initial sporophytes, were conducted for each tested light quality.

The analysis of photosynthetic pigments was conducted after 2 months of the culture of whole sporophytes. Moreover, analysis of phenolic acids and flavonoids was performed for plant extracts prepared from initial sporophytes (etiolated, with 3–4-leaves) of *C. delgadii*, plants obtained after 6-month-long culture under fluorescent lamps and LEDs, and the mature plants (both aerial and underground parts) growing in the greenhouse of the University of Warsaw Botanic Garden (Warsaw, Poland).

### 2.2. Evaluation of Regenerative Ability

In the *C. delgadii* stipe explants, the influence of light conditions on the efficiency of sporophytogenesis and gametophytogenesis as well as the sporophyte development were measured after 8 weeks of culture, while the ability of whole etiolated sporophytes to multiplicate and produce new plants was described after 6 months of the culture. The analysed parameters with methods of their calculation are summarised in Table 1. 

The visualisation of regeneration ability was made using stereo microscope (Olympus SZH, Tokyo, Japan) and photographed by camera (Olympus SC30, Tokyo, Japan) with conducted computer program (cellSens Standard).

### 2.3. Content of Photosynthetic Pigments

The level of photosynthetic pigments was measured with a spectrophotometric method. Cut leaf samples (200 mg) were homogenised in a mortar and dissolved in 80% acetone. After filtration, the absorbance was measured using a UV/VIS Helios Alpha spectrophotometer (Unicam Ltd., Cambridge, UK). The absorbance was measured at the following wavelength maxima: 663.2 nm, 646.8 nm, and 470 nm for chlorophyll *a*, chlorophyll *b*, and carotenoids, respectively. The content of chlorophyll *a*, chlorophyll *b*, and total carotenoids was calculated according to the methodology of Lichtenthaler and Buschmann [42] and total chlorophyll according to the work of Khasanah and Maryani [43]. Tree biological replicates were processed per light condition.

### 2.4. Secondary Metabolite Extraction

The plants (both aerial and underground parts) were frozen (−80 °C) and extracted by sonication at controlled temperature (40 ± 2 °C) for 30 min with a mixture of methanol–acetone–water (3:1:1, *v*/*v*/*v*; 3 × 50 mL). The combined extracts were then filtered, evaporated under reduced pressure, and lyophilised in a vacuum concentrator (Free Zone 1 apparatus; Lab-conco, Kansas City, MO, USA) to obtain dried residues. Six biological replicates were processed per light condition.

### 2.5. LC-ESI-MS/MS Analysis of Phenolic Acids and Flavonoids

The contents of phenolic acids and flavonoids were analysed by high-performance liquid chromatography coupled with electrospray ionisation mass spectrometry (LC-ESI-MS/MS), using the slightly modified method previously described by Pietrzak et al. [44]. An Agilent 1200 Series HPLC system (Agilent Technologies, Santa Clara, CA, USA) connected to a 3200 QTRAP Mass spectrometer (AB Sciex, Redwood City, CA, USA) with electrospray ionisation source (ESI) operating in negative-ion mode were used for all analytes. Both were controlled with Analyst 1.5 software (AB Sciex, Framingham, MA, USA), which was also used for data interpretation. 

Separation of phenolics was carried out at 25 °C, on a Zorbax SB-C18 column (2.1 × 100 mm, 1.8 mm particle size; Agilent Technologies, Santa Clara, CA, USA). The mobile phase consisted of 0.1% aqueous formic acid (solvent A) and acetonitrile with 0.1% formic acid (solvent B). The injection volume was 3 µL, and the flow rate was 300 µL/min. The gradient was changed as follows: 0–2 min—20% B; 3–4 min—25% B; 5–6 min—35% B; 8–12 min—65% B; 14–16 min—80% B; and 20–28 min −20% B.

The ESI-MS worked in negative ion mode, and the parameters were: capillary temperature 450 °C, curtain gas at 30 psi, nebuliser gas at 50 psi, and source voltage−4500 V for determination of phenolic acids and flavonoid compounds. 

Triplicate injections were made for each standard solution and sample. The limits of detection (LOD) and quantification (LOQ) for all analytes were determined at a signal-to-noise ratio of 3:1 and 10:1, respectively. Qualitative identification of compounds was done by comparison of the MS/MS spectra and LC retention time with the corresponding standards tested under the same conditions. The calibration curves obtained in MRM mode were used for quantification of all analytes.

### 2.6. Antioxidant Activity Assays

All analyses were done using 96-well microplates (Nunclon, Nunc, Roskilde, Denmark) and were measured in an ELISA Reader Infinite Pro 200F (Tecan Group Ltd., Männedorf, Switzerland).

Scavenging of DPPH^●^ (2,2-diphenyl-1-picryl-hydrazyl) radical was measured using method of Olech et al. [45]. The absorbance was controlled at 517 nm after 30 min incubation at 28 °C. Ascorbic acid was used as a positive control. 

Scavenging of ABTS^●+^ (2,2′-azinobis [3-ethylbenzthiazoline]-6-sulfonic acid) was examined using previously described method [45]. The absorbance was controlled at 734 nm. Ascorbic acid was used as a positive control.

The metal chelating activity was evaluated by the method described by Guo and co-authors [46] and modified by Szewczyk et al. [47]. The absorbance was controlled at 562 nm. EDTA (ethylenediaminetetraacetic acid) was used as a positive control.

Antioxidant activity was also estimated using the β-carotene bleaching method described previously by Deba et al. [48]. The absorbance was controlled at 470 nm. Butylated hydroxytoluene (BHT) was used as a positive control.

The antioxidant capacity in all performed assays was expressed as EC_50_, which is the concentration of extract or standard required to achieve 50% antioxidant activity. Each assay was repeated three times.

### 2.7. Statistical Analysis

Data were analysed statistically using Statistica version 13 and PQStat software. When data fulfilled the assumptions of normality and homoscedasticity, an analysis of variance (ANOVA) was performed followed by the Duncan’s post hoc multiple range test (at *p* ≤ 0.05). When data were not normally distributed, a Kruskal–Wallis test was performed followed by Dunn–Bonferroni post hoc test to identify significant differences (at *p* ≤ 0.05).

## 3. Results

### 3.1. Efficiency of Sporophyte and Gametophyte Production on the Stipe Explants

**Figure 1:**
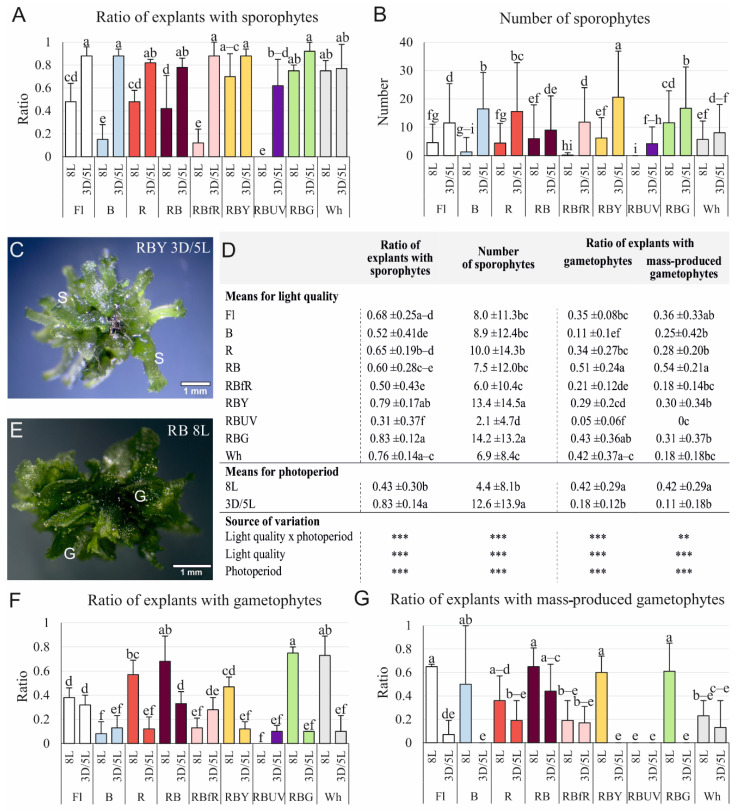
The influence of light quality and photoperiod on the efficiency of sporophytogenesis and gametophytogenesis after 8 weeks of culture of the *Cyathea delgadii* stipe explants. (**A**) The ratio of explants with sporophytes and (**B**) the number of sporophytes. (**C**) The picture of juvenile sporophytes developed after 3D/5L treatment under RBY LED light. (**D**) Separation of significantly different mean values of described parameters for light quality and photoperiod used in the cultures. (**E**) Mass gametophyte production after culture under RB LED light (8L). (**F**) The ratio of explants with gametophytes and (**G**) mass-produced gametophytes. Fl—control light: fluorescence Philips TL-D 36W/54 lamps; B—100% blue LED light (430 nm); R—100% red LED light (670 nm); RB—combination of red (70%) and blue (30%) LED lights; RBfR—combination of red, blue, and far-red (730 nm) LED lights (35%/15%/50%); RBY—combination of red, blue, and yellow (600 nm) LED lights (35%/15%/50%); RBUV—combination of red, blue, and UV (400 nm) LED lights (35%/15%/50%); RBG—combination of red, blue, and green (528 nm) LED lights (35%/15%/50%); Wh—white LED (1:1:1 2700K:4500K:5700K); 8L—8-week-long culture under 16/8 h light/dark; 3D/5L—5-week-long culture under 16/8 h light/dark preceded by 3-week-long culture under 24 h dark; Mean ± standard deviations marked by different letters are significantly different (*p* ≤ 0.05) following Duncan’s multiple range test; Significant effect: ** *p* ≤ 0.05; *** *p* ≤ 0.01; G—gametophyte, S—sporophyte.

The light conditions, under which the cultures of the *C. delgadii* stipe explants were initiated, affected the efficiency of the sporophyte and gametophyte production (Figure 1). The ratio of explants with sporophytes was similar for eight of the nine tested light qualities when explants were cultured with a 3-week-long dark period (3D/5L). In the case of the cultures without this period (8L), the stimulatory effect on this parameter was revealed for RBG and Wh lights (Figure 1A). Under RBY light (3D/5L), the number of sporophytes produced was the greatest (equal to 20.6) (Figure 1B,C). A statistical analysis revealed that the ratio of explants with sporophytes and the number of sporophytes per explant were two and three times greater, respectively, under 3D/5L photoperiod conditions in comparison to photoperiod 8L. Moreover, RBY and RBG lights stimulated sporophytogenesis regardless of the photoperiod conditions (Figure 1D).

Gametophytes were the most frequently obtained in the cultures under photoperiod 8L and RB, RBG and Wh lights (Figure 1D–F). The mass production of gametophytes occurred under six of the nine tested light spectra, i.e., Fl, B, R, RB, RBY, and RBG (Figure 1G). A statistical analysis confirmed that RB light was the most suitable for the gametophyte formation with high effectiveness (Figure 1D,E).

### 3.2. Effect of Light Quality and Photoperiod on the Development of Sporophytes Obtained on the Stipe Explants

**Figure 2:**
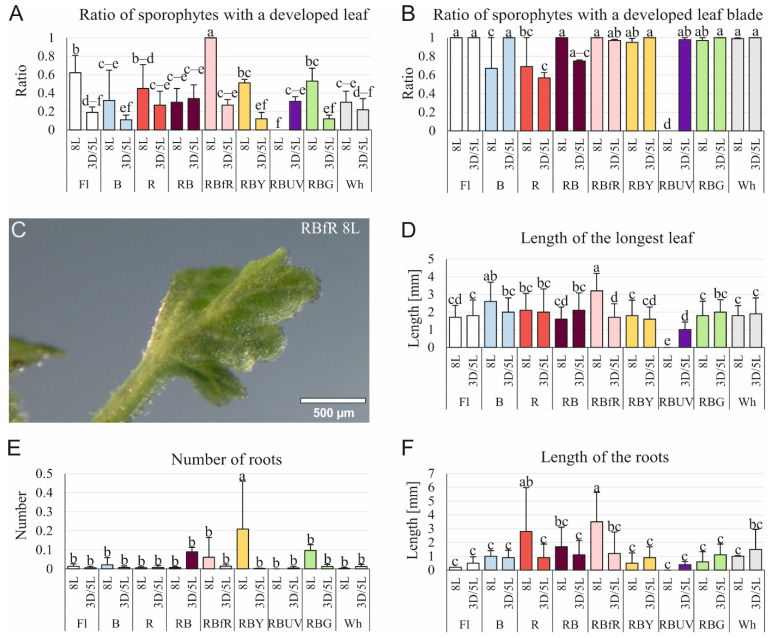
The influence of light conditions on the sporophyte development of *Cyathea delgadii* after 8-week-long culture of stipe explants. (**A**) The ratio of sporophytes with a developed leaf and (**B**) developed leaf blade. (**C**) Picture of well-developed leaf blade obtained under RBfR LED light (8L). (**D**) Length of the longest leaf. (**E**) Root production. (**F**) Length of the roots. Fl—control light: fluorescence Philips TL-D 36W/54 lamps; B—100% blue LED light (430 nm); R—100% red LED light (670 nm); RB—combination of red (70%) and blue (30%) LED light; RBfR—combination of red, blue, and far-red (730 nm) LED lights (35%/15%/50%); RBY—combination of red, blue, and yellow (600 nm) LED lights (35%/15%/50%); RBUV—combination of red, blue and UV (400 nm) LED lights (35%/15%/50%); RBG—combination of red, blue, and green (528 nm) LED lights (35%/15%/50%); Wh—white LED (1:1:1 2700K:4500K:5700K); 8L—8-week-long culture under 16/8 h light/dark; 3D/5L—5-week-long culture under 16/8 h light/dark preceded by 3-week-long culture under 24 h dark; Mean ± standard deviations marked by different letters are significantly different (*p* ≤ 0.05) following Duncan’s multiple range test.

Light conditions affected the biometrical properties of the *C. delgadii* sporophytes (Figure 2). The greatest ratio of sporophytes with a developed leaf blade was obtained under the RBfR light spectrum in the culture without a 3-week-long dark period (Figure 2A). The lowest ratio of sporophytes with a developed leaf blade was obtained under RBUV light (8L) (Figure 2B); however, most of the tested light conditions had a positive influence on this parameter (Figure 2B,C). The RBfR light (8L) promoted the elongation of leaves. Under this light, the mean length of the longest leaf was equal to 3.2 mm (Figure 2D). The number of roots was stimulated by the RBY light spectrum (8L) (Figure 2E). The mean length of the roots under RBfR (8L) light was enhanced in comparison to most of the other light conditions and equalled 3.5 mm (Figure 2F).

### 3.3. Effect of Light Quality on the Sporophyte Production on Whole Etiolated Sporophytes

**Figure 3:**
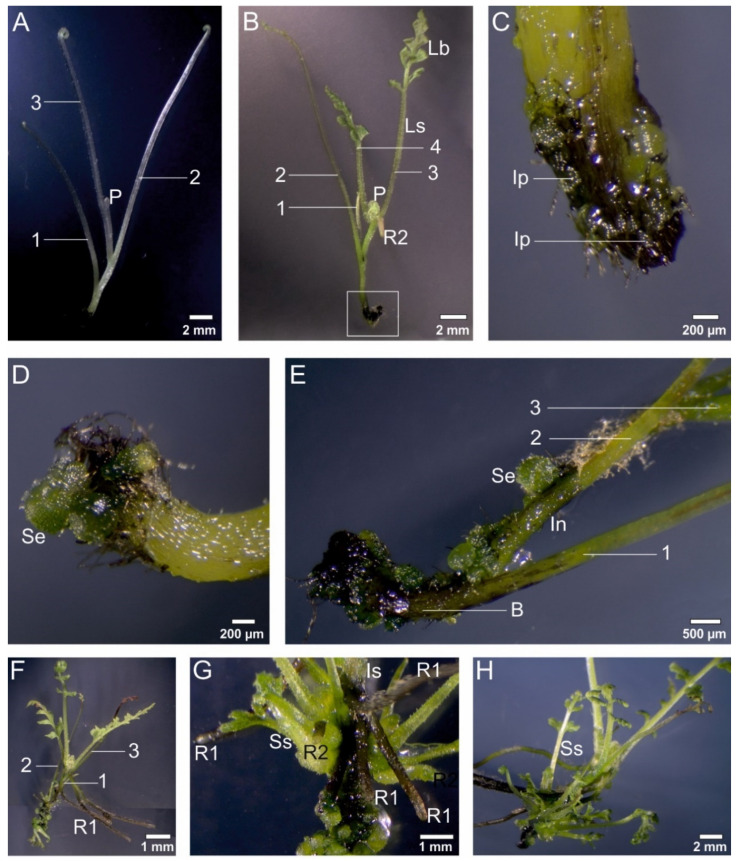
The proliferation of new sporophytes via somatic embryogenesis on whole etiolated 5-month-old sporophytes of *Cyathea delgadii* during culture under 16/8 h photoperiod conditions under fluorescent light. (**A**) initial sporophyte with reduced leaf blades. (**B**) Sporophyte with well-developed leaf blades after 3 weeks of the culture. (**C**) Intensive proliferation at the base of the initial sporophyte, and embryo structures protruding above its surface (magnification of the picture (**B**) after 3 weeks of the culture. (**D**) Somatic embryos at the base of initial sporophyte after 4 weeks of the culture. (**E**) Somatic embryos formed at the base of sporophyte and on the internode between 1st and 2nd leaf after 5 weeks of the culture. (**F**) Long roots of initial sporophytes (R1) after 6 weeks of the culture. (**G**) Somatic embryo-derived sporophytes with differentiated roots (R2) after 7 weeks of the culture. (**H**) Well-developed somatic embryo-derived sporophytes after 8 weeks of the culture. 1—first leaf, 2—second leaf, 3—third leaf, 4—fourth leaf, B—base of sporophyte, Ip—intensive proliferation, In—internode, Is—initial sporophyte, Lb—leaf blade, Ls—leaf stipe, P—leaf primordium, R1—root of initial sporophyte, R2—root of somatic embryo-derived sporophyte, Se—somatic embryo, Ss—somatic embryo-derived sporophyte.

When whole etiolated 5-month-old sporophytes of *C. delgadii* (Figure 3A) were moved to a fresh medium and cultured under 16/8 h photoperiod conditions using four light combinations (Fl, B, R, and RB), their leaves developed and formed a typical leaf blade (Figure 3B). Their development was accompanied by the proliferation of cells and formation of new sporophytes. 

After 3 weeks of culture, intensive proliferation was visible at the base of these sporophytes (Figure 3C). One week later, somatic embryos were observed at those locations (Figure 3D). After 5 weeks of culture, the somatic embryos were also formed on the internodes located between the first and second leaf of the sporophytes (Figure 3E). Long roots of the initial sporophytes were abundantly observed (Figure 3F). The somatic embryo-derived juvenile sporophytes rapidly developed, and their roots and well-developed leaf blades were noticed after 7 and 8 weeks of culture, respectively (Figure 3G,H).

**Figure 4:**
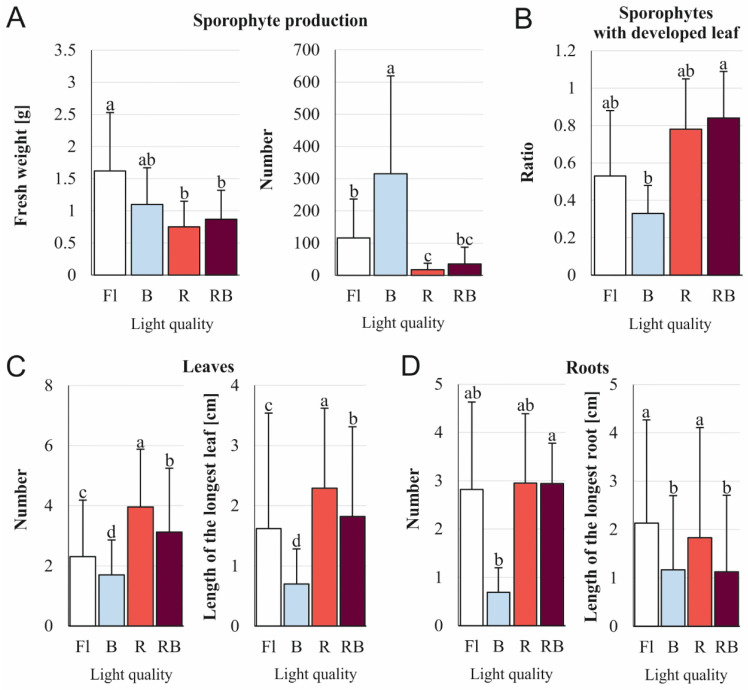
Effect of different light quality on fresh weight and biometrical properties of growth of *Cyathea delgadii* sporophytes after in vitro propagation under: Fl—fluorescent light; B—100% blue LED light; R—100% red LED light; RB—combination of red (70%) and blue (30%) LED light. The effect on 5-month-old etiolated sporophytes was estimated after 6-month-long culture. (**A**) Fresh weight of proliferated plants and number of obtained sporophytes. (**B**) Ratio of sporophytes with a developed leaf. (**C**) Number of leaves and length of the longest leaf. (**D**) Number of roots and length of the longest root. Mean ± standard deviations marked by different letters are significantly different (*p* ≤ 0.05) following Dunn–Bonferroni test.

The light quality had an influence on the proliferation of new sporophytes and their development (Figure 4). After 6 months of culture, there was no statistical difference between the fresh weight of plants obtained under control Fl light (1.62 g) and B light (1.1 g). In the B light spectrum, the number of newly-formed sporophytes per initial sporophyte was the greatest (315.2), and it was 2.7, 9, and 18.2 times greater than under the Fl, RB, and R lights, respectively (Figure 4A). The B, R, and RB lights did not affect the development of leaves as compared to the Fl light (Figure 4B). The number and the length of leaves were stimulated by the R light (Figure 4C). The tested LEDs had no influence on the number of roots in comparison to the Fl light. The length of the root was stimulated by the Fl and R lights and was equal to 2.13 and 1.83 cm, respectively (Figure 4D).

### 3.4. Photosynthetic Pigment Content

**Figure 5:**
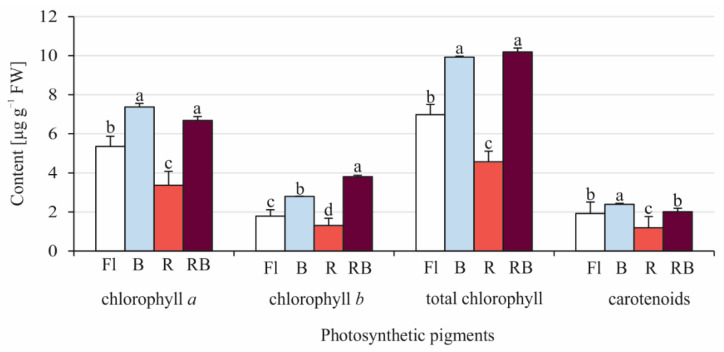
Effect of different light quality on the content [μg g^−1^ fresh weight ± SD] of photosynthetic pigments of *Cyathea delgadii* after multiplication in vitro under: Fl—fluorescent light; B—100% blue LED light; R—100% red LED light; RB—combination of red (70%) and blue (30%) LED light; data are means. Shared letters within each pigment indicate no statistically significant difference following Duncan’s multiple range test at *p* ≤ 0.5.

The photosynthetic pigment contents varied depending on the light quality under which the sporophytes developed (Figure 5). The production of *a*, *b*, and total chlorophyll was the greatest under the B and RB light spectra. Under all tested light qualities, carotenoids were accumulated in lower quantities than total chlorophyll. The greatest carotenoid content was observed under the B light spectrum, and it was equal to 2.39 μg g^−1^ fresh weight. The R light used alone was ineffective for the accumulation of all photosynthetic pigments studied here.

### 3.5. Secondary Metabolite Profiles

To describe the influence of different light conditions on the accumulation of the potentially active compounds in the *C. delgadii* tissues, the LC-ESI-MS/MS analysis of their extracts was conducted. Only 11 of the 23 analysed compounds were found in the extracts at a content equal to or higher than the limit of quantification (Table 2). The rest of the analysed secondary metabolites are listed in Supplementary Table S1. The plants cultured under F light were characterised by the greatest content of the five tested compounds (i.e., protocatechuic acid, *cis*-5-*O*-caffeoylquinic acid, caffeic acid, quercitrin, and naringenin 7-*O*-glucoside). In turn, the plants growing under the B light spectrum had the greatest content of four compounds (i.e., rutin, isoquercetin, nicotiflorin, and astragalin). The content of *trans*-5-*O*-caffeoylquinic acid was the greatest under RB light (1.0129 µg/mg of dry weight). It was the most abundant metabolite found in the extracts. Among all the tested flavonoid aglycones, only prunetin was detected. Plants from the greenhouse accumulated the lowest number of tested secondary metabolites (only three) and at a low content.

### 3.6. Antioxidant Activity of Plants Extracts

The extracts obtained from *C. delgadii* growing in the greenhouse and under Fl light had the ability to neutralise the DPPH^●^ free radical comparable to ascorbic acid used as a positive control (Table 3). In turn, these two extracts had a moderate ability to scavenge the ABTS^●+^ radical cation, since the EC_50_ values were lower than for ascorbic acid; however, these were the best results among all the tested extracts. Each tested extract had a worse ferrous chelating effect in comparison to EDTA (positive control). In the carotene bleaching protocol, the extract from plants growing in the greenhouse inhibited the oxidation of linoleic acid the most (EC_50_ = 22.68 mg/mL).

## 4. Discussion

### 4.1. Effect of Light Conditions on the Production of Somatic Embryo-Derived Plantlets

Our previous research showed that light conditions, in which *C. delgadii* plants grow in vitro, regulate the regenerative ability of explants excised from these plants. To trigger embryogenic competence of explants, a long-term culture of donor plants in constant darkness is required [30]. This treatment changes the hormonal status of stipe explants and allows their cells to form somatic embryos [35]. Etiolated *C. delgadii* plants are capable of SE in both dark and light [33]. In this research, we showed that a period of darkness at the beginning of the culture of *C. delgadii* stipe explants enhanced SE. Similarly, the positive effect of the culture conducted in the dark has been previously shown for the production of somatic embryos of *Pelargonium x hortorum* Bailey and *Coffea arabica* [49,50].

The number of studies testing the influence of a wide range of light spectra on induction of SE is limited. In our study, we indicated that the ratio of explants able to form somatic embryos is high under RBY and RBG light, and under these two lights, the number of sporophytes formed by the explant cells of *C. delgadii* was the highest, regardless of the photoperiod conditions (Figure 1D). In turn, the combination that enhanced the highest total number of somatic embryos obtained from callus of *Peucedanum japonicum* consisted of red and blue lights [8], while the combination of red and far-red lights was the most efficient for somatic embryo production in *Doritaenopsis* hybrid [51]. These results indicate that the light spectrum may cause a different embryogenic response depending on the plant species or genotype.

The excision of the explant is not necessary to induce SE in *C. delgadii*. It has been previously shown that whole etiolated plants are able to develop somatic embryos when maintained in an aging culture [30]. In the present study, our analysis of the influence of light on initial sporophytes showed that the weight of multiplied plants was not statistically different under the control Fl light and B light. In turn, the use of fluorescent lamps in *Withania* *somnifera* cultures increased the fresh weight of propagated plants when compared to the tested LED lights [52]. The B light promoted the number of new somatic-embryo-derived sporophytes of *C. delgadii*. This light also had a positive impact on the direct SE of *Dianthus caryophyllus* [53].

### 4.2. Effect of Light Conditions on Plant Development

#### 4.2.1. Leaves

In *C. delgadii*, a 3-week-long period of darkness at the beginning of the culture resulted in reduced development of leaves. It is in line with the results obtained for *Lippia alba* [54]. Most of the tested light combinations resulted in similar ratios of sporophytes with a developed leaf blade, excluding R light that significantly inhibited this parameter. As previously shown, red light may inhibit the mesophyll tissue thickness and leaf density [55]. This light may cause the collapse of mesophyll cells and a reduction in leaf blades [56]. In *C. delgadii*, however, the number of leaves under R light was greater than under Fl, RB, and B lights (Figure 4C). A similar effect of red light was also observed in *Taraxacum officinale* and *Protea cynaroides* [57,58].

The 8-week-long culture of *C. delgadii* stipe explants under B light had a better impact on leaf elongation in comparison to Fl light (Figure 2D); however, an opposite effect of this light on the multiplication of somatic embryos on the whole initial sporophytes was observed (Figure 4C). Similarly, Santos Lazzarini et al. [59] demonstrated various responses of two types of *Lippia gracilis* explants, namely, apical and nodal segments, under light treatments. Thus, our results provide evidence that the influence of a specific light spectrum depends on the type of plant material used in a culture.

#### 4.2.2. Roots

Research on the influence of light on plants propagated in in vitro cultures has shown not only the impact on biometric parameters of leaves, but also on roots. In nature, roots grow in the dark since the light penetrate less than 4 mm through the soil [60]; however, roots of plants growing in transparent media are directly illuminated [61]. The development of root systems in laboratory conditions usually enhances the survival rate in the subsequent acclimatisation to the natural environment [62]. Light induces root growth by providing sugars and auxin transported from aerial parts of plants [63,64]. The production of the roots by *C. delgadii* sporophytes was the most efficient under RBY light. In turn, Su et al. [65] showed the opposite effect of yellow light for root formation in *Cucumis sativus*. The root elongation of *C. delgadii* was the most stimulated by RBfR and R lights. Similar to our study, the elongation of roots under a combination of red, blue, and far-red light was observed in *Oncidium* plantlets [66], while elongation under red light was noticed in *T. officinale* and *P. amboinicus* [57,67].

### 4.3. Effect of Light Conditions on Fern Gametophyte Production

The culture conditions for gametophyte formation are important for effective fern propagation because the gametophyte is the most sensitive stage in the life cycle of ferns [68]. Knowledge about the influence of different light qualities on the process of gametophytogenesis is lacking. Similar to studies on *C. delgadii*, the darkness inhibited the formation of gametophytes of *C. corcovadensis* [36] and *Adiantum reniforme* var. *sinense* [68]. Previous studies indicated that the gametophytes of fern *Adiantum capillus-veneris* contain blue-light receptors and a red-light receptor [69,70]. Red and blue lights could have an impact on the shape of gametophytes [71] as well as on the shape of their cells [37]. These findings are consistent with our results since RB light was beneficial for gametophyte production. Furthermore, our research indicated that gametophyte production of *C. delgadii* was also favoured by RBG light. However, the growth of gametophytes was very slow in green light in *Pteridium aquilinum* [72].

Previous studies demonstrated that UV light had a negative effect on the growth of the gametophytes of *Cheilanthes rufa* and *Acrostichum danaeifolium* [73,74]. Such results were observed in our study when *C. delgadii* explants were cultured under RBUV light.

### 4.4. Effect of Light Quality on Content of Photosynthetic Pigments

The content of photosynthetic pigments in *C. delgadii* was dependent on the tested light conditions. We indicated that both B and RB lights were the best for chlorophyll biosynthesis, while R light was not effective in the accumulation of any photosynthetic pigments. The results are consistent with studies showing that plants grown under blue light had a more compact shape and a higher chlorophyll content at the same time in comparison to plants grown under red light [75,76,77]. It has been reported that an accumulation of photosynthetic pigments was lower in plants of *Gerbera jamesonii* and *Myrtus communis* multiplied under R light from the same LED panel as used in our research [9,78]. Plants with a high chlorophyll content are more resistant to abiotic stresses, which positively impacts their yield [79]. The chlorophyll content was correlated with the thickness of the spongy mesophyll of leaf blades in gerbera [80]. According to Liu et al. [81], the chloroplasts of *Solanum lycopersicum* were well developed under blue light, while red light caused their dysplasia. Blue light enhanced the biosynthesis of chlorophyll precursors in Chinese cabbage [75]. Miao et al. [82] showed that this light plays a role in maintaining photosystem I and II activity and photosynthetic electron transport capacity in cucumber. The results for *C. delgadii* showed that B light was also the most effective for the accumulation of carotenoids. Similarly, the results obtained by Simlat et al. [83] in *Stevia rebaudiana* and by Zheng and van Labeke [84] in *Sinningia speciosa* cultures showed that the accumulation of carotenoids was stimulated by the blue light spectrum.

### 4.5. Effect of Light Quality on Secondary Metabolite Production and Their Antioxidant Activity

Secondary metabolites are compounds that play an important role in the interaction of plants with their environment [85]. Therefore, it is not surprising that their biosynthesis in plant tissues is modified by changing environmental conditions including light [86]. In our research, the secondary metabolite with the highest content in *C. delgadii* tissues was 5-*O*-caffeoylquinic acid (often referred to as chlorogenic acid). This acid is known for its wide range of beneficial properties for human health, including anti-inflammatory, antioxidant, antimicrobial, anticancer, and neuroprotective [87]. In *C. delgadii* plants, B light was the most efficient for the accumulation of flavonoid glycosides, such as astragalin and rutin. A stimulatory effect of this light quality on the accumulation of secondary metabolites was also noticed for other species, e.g., *Rhodiola imbricate* [88] and *Capsicum annuum* [89]. Wang et al. [90] indicated that blue light-responsive genes were related to flavonoid synthesis in tea plants.

Some of the secondary metabolites identified in *C. delgadii* tissues were also noticed in other species belonging to *Cyathea* genus. Astragalin was accumulated by *C. fauriei*, *C. martensiana*, *C. leichhardtiana*, *C. podophylla,* and *C. hancockii* [91]. Moreover, the last two species accumulated nicotiflorin [91], while rutin was identified in *C. crinita* [18].

Environmental stresses, such as pathogen attacks and UV-B radiation, led to an enhancement of reactive oxygen species (ROS) production in plants. Excessive ROS generation causes oxidative damage of cells leading to their death. Antioxidant agents, including enzymatic (e.g., superoxide dismutase) and nonenzymatic components (e.g., phenolic compounds and carotenoids) can help to overcome this oxidative damage [92]. In our research, extracts from plants growing in the greenhouse had the best antioxidant activity, probably linked to increased tolerance of these plants to environmental stresses. Taking into consideration that *C. delgadii* plants cultured under the B light spectrum were rich in secondary metabolites and weak in antioxidant activity, we supposed that antioxidant activity of this species may relate to some phenolics not analysed in our research or with enzymatic scavengers; however, further research is needed to confirm this.

## 5. Conclusions

**Figure 6:**
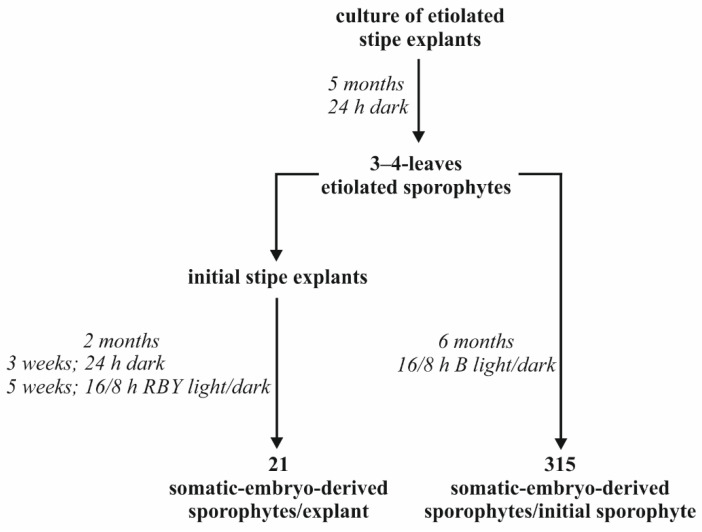
The best conditions for mass production of *Cyathea delgadii* sporophytes.

These studies confirm that light is a critical factor for in vitro cultures of the tree ferns since it affects the growth and development of the *C. delgadii* plants as well as their photosynthetic pigments and secondary metabolite accumulation. Our studies indicate that a period of darkness applied for the induction of SE in fern contributes to a high production of new sporophytes and a low production of gametophytes. The influence of light quality on plant development depends on the type of plant material used for the culture induction. The RBY and B lights were found to be the most efficient for a high production of somatic embryo-derived sporophytes when stipe explants and whole young sporophytes were cultured, respectively (Figure 6). Moreover, root production is stimulated by RBY light. The RBfR light is effective for the elongation of roots and leaves and for the development of leaves. The RB light is the best light quality for gametophyte mass production. The RB and B lights stimulate the accumulation of chlorophyll in fern sporophytes better than Fl light. The Fl, B, and RB lights are effective for the accumulation of secondary metabolites. The results are helpful for the improvement of micropropagation conditions of ferns and show that metabolite accumulation can be controlled by different light spectra.

## Figures and Tables

**Table 1:** The parameters used for evaluation of regenerative ability of stipes and whole sporophytes of *Cyathea delgadii*.

Parameter	Method of Calculation
Experiment with Stipe Explants	
Ratio of explants with sporophytes	the number of explants with regenerated sporophytes divided by the total number of stipe explants used for culture initiation
Number of sporophytes	the total number of obtained sporophytes divided by the sum of cultured explants
Ratio of explants with gametophytes	the number of explants with regenerated gametophytes divided by the total number of explants used for culture initiation
Ratio of explants with mass-produced gametophytes	the number of explants that produced gametophytes with total surface equal or higher than 4 mm^2^, per total number of explants with gametophytes
Ratio of sporophytes with a developed leaf	the number of sporophytes with at least 1 leaf at crosier stadium or older per sum of all sporophytes (including these with only a leaf primordium)
Ratio of sporophytes with a developed leaf blade	the number of sporophytes with at least 1 leaf with the leaf blade older than crosier stadium per total number of sporophytes with a developed leaf
Length of the longest leaf	the sum of the length of the longest leaf from each sporophyte per the sum of obtained sporophytes
Number of roots	the sum of roots divided by the total number of sporophytes
Length of the roots	the sum of root length divided by the total number of sporophytes
Experiment with whole sporophytes	
Fresh weight	weight of total proliferated plants obtained from a single initial sporophyte
Number of sporophytes	the sum of newly-formed sporophytes per the total number of initial sporophytes
Ratio of sporophytes with a developed leaf	the sum of sporophytes with at least 1 leaf at crosier stadium or older per sum of all sporophytes (including these with only a leaf primordium)
Number of leaves	the sum of leaves produced by sporophytes divided by the total number of sporophytes
Length of the longest leaf	the length of the longest leaf of each sporophyte divided by the total number of sporophytes
Number of roots	the sum of roots divided by the total number of sporophytes
Length of the longest root	the sum of the length of the longest root of each sporophyte divided by the total number of sporophytes

**Table 2:** Content of phenolic acids, flavonoid aglycones, and flavonoid glycosides in the extracts from *Cyathea delgadii*, growing under various light conditions.

Compound	Retention Time [min]	[M-H]- [*m*/*z*]	Fragment Ions [*m*/*z*]	Collision Energy [eV]	Light Conditions					
					Dark	Fl	B	R	RB	Greenhouse
					Compound Content [µg/mg of Dry Weight]					
Phenolic acids										
Protocatechuic acid	8.46	152.9	80.9 107.8	−26 −38	0.0058 ± 0.0002 d	**0.0098** ± 0.0006 a	0.0048 ± 0.0001 e	0.0067 ± 0.0006 c	0.0076 ± 0.0003 b	0.0033 ± 0.0001 f
*Trans*-5-*O*-caffeoylquinic acid	9.32	352.9	190.8 84.9	−24 −60	0.3698 ± 0.0075 e	0.7004 ± 0.0064 c	0.7420 ± 0.0562 b	0.5733 ± 0.0143 d	**1.0129** ± 0.0306 a	0.0028 ± 0.0003 f
*Cis*-5-*O*-caffeoylquinic acid	10.45	352.9	190.8 84.9	−24 −60	0.0940 ± 0.0065 d	**0.2361** ± 0.0140 a	0.0413 ± 0.0014 e	0.1268 ± 0.0043 b	0.1073 ± 0.0049 c	0.0006 ± 0.0001 f
Caffeic acid	11.40	178.7	88.9 134.9	−46 −16	0.0019 ± 0.0002 b	**0.0037** ± 0.0001 a	nd	0.0012 ± 0.0001 c	nd	nd
Flavonoid aglycones										
Prunetin	21.98	282.8	267.7 238.7	−20 −26	nd	**0.00017** ± 0.000005 c	**0.00020** ± 0.00002 b	**0.00016** ± 0.000004 d	**0.00024** ± 0.000015 a	nd
Flavonoid glycosides										
Quercetin 3-*O*-rutinoside (Rutin)	11.99	608.7	299.6 270.9	−46 −60	BQL	0.0346 ± 0.0028 c	**0.0603** ± 0.0005 a	0.0081 ± 0.0001 d	0.0571 ± 0.0004 b	nd
Quercetin 3-*O*-glucoside (Isoquercetin)	13.00	462.7	299.7 270.7	−30 −44	nd	0.0327 ± 0.0022 c	**0.1142** ± 0.0034 a	BQL	0.0483 ± 0.0009 b	nd
Kaempferol 3-*O*-rutinoside (Nicotiflorin)	13.31	592.7	284.8 226.7	−38 −68	0.0005 ± 0.00003 e	0.0476 ± 0.0011 c	**0.0742** ± 0.0020 a	0.0273 ± 0.0017 d	0.0686 ± 0.0021 b	nd
Kaempferol 3-*O*-glucoside (Astragalin)	14.66	446.7	226.8 254.8	−54 −40	nd	0.0360 ± 0.0004 b	**0.0728** ± 0.0039 a	BQL	0.0316 ± 0.0011 c	BQL
Quercetin 3-*O*-rhamnoside (Quercitrin)	14.83	446.7	299.7 270.7	−30 −40	BQL	**0.0091** ± 0.0009 a	0.0049 ± 0.0003 b	BQL	0.0021 ± 0.0002 c	nd
Naringenin-7-*O*-glucoside	15.12	432.7	270.8 118.9	−22 −64	0.0007 ± 0.00004 d	**0.0183** ± 0.0004 a	0.0144 ± 0.0005 b	0.0136 ± 0.0005 c	0.0143 ± 0.0006 b	nd

Mean ± standard deviations marked by different letters are significantly different (*p* ≤ 0.05) within each secondary metabolite following Duncan’s multiple range test at (*p* ≤ 0.5).; *n* = 6; nd—not detected; BQL—peak detected, concentration higher than the limit of detection but lower than the limit of quantification. The highest content of each secondary metabolite is bold. Fl—control light: fluorescence Philips TL-D 36W/54 lamps; B—100% blue LED light (430 nm); R—100% red LED light (670 nm); RB—combination of red (70%) and blue (30%) LED light. Cultures were maintained under a 16/8 h light/dark.

**Table 3:** The antioxidant activity of the *Cyathea delgadii* extracts from plants growing under various light conditions.

Antioxidant Activity	Light Conditions					
	Dark	Fl	B	R	RB	Greenhouse
DPPH (EC_50_ mg/mL)	0.72 ± 0.78 ab	0.08 ± 0.07 a	0.88 ± 0.14 b	0.39 ± 0.20 ab	0.36 ± 0.39 ab	0.03 ± 0.01 a
ABTS (EC_50_ mg/mL)	1.87 ± 0.13 cd	0.59 ± 0.10 a	2.24 ± 0.50 d	1.43 ± 0.25 bc	1.32 ± 0.17 b	0.35 ± 0.15 a
CHEL (EC_50_ mg/mL)	1.65 ± 0.15 b	1.42 ± 0.12 b	1.15 ± 0.11 a	1.50 ± 0.10 b	2.15 ± 0.20 c	2.70 ± 0.18 d
β-Carotene/linoleic acid (EC_50_ μg/mL)	52.10 ± 0.16 d	31.70 ± 0.12 b	72.45 ± 0.75 e	32.17 ± 0.20 b	34.85 ± 0.13 c	22.68 ± 0.39 a

Fl—control light: fluorescence Philips TL-D 36W/54 lamps; B—100% blue LED light (430 nm); R—100% red LED light (670 nm); RB—combination of red (70%) and blue (30%) LED light. Cultures were maintained under a 16/8 h light/dark; EC_50_ value is the concentration of extract or standard required to achieve 50% antioxidant activity. Each value is expressed as mean ± standard deviation (*n* = 3); DPPH (2,2-diphenyl-1-picryl-hydrazyl) assay for ascorbic acid (standard) EC_50_ = 0.05 ± 0.02 mg/mL; ABTS (2,2′-azinobis[3 -ethylbenzthiazoline]-6-sulfonic acid) assay for ascorbic acid (standard) EC_50_ = 0.12 ± 0.10 mg/mL; metal chelating activity (CHEL) for ethylenediaminetetraacetic acid (EDTA) (standard) EC_50_ = 0.63 ± 0.10 mg/mL; β-carotene bleaching assay for butylated hydroxytoluene (BHT) (standard) EC_50_ = 32.15 ± 0.20 μg/mL.; mean ± standard deviations marked by different letters are significantly different (*p* ≤ 0.05) following Duncan’s multiple range test.

## Data Availability

The data sets generated for this study are available on request from the corresponding author.

## Supplementary Materials

The following supporting information can be downloaded at: https://www.mdpi.com/article/10.3390/cells11030486/s1, Table S1. The list of secondary metabolites analysed in the extracts from *Cyathea delgadii*, growing under various light conditions, but not detected or at a concentration higher than the limit of detection but lower than the limit of quantification.

## References

[1] Dong C., Fu Y., Liu G., Liu H. (2014). Low light intensity effects on the growth, photosynthetic characteristics, antioxidant capacity, yield and quality of wheat (*Triticum aestivum* L.) at different growth stages in BLSS. Adv. Space Res..

[2] Huang X., Ouyang X., Deng X.W. (2014). Beyond repression of photomorphogenesis: Role switching of COP/DET/FUS in light signaling. Curr. Opin. Plant Biol..

[3] Bula R.J., Morrow R.C., Tibbitts T.W., Barta D.J., Ignatius R.W., Martin T.S. (1991). Light-emitting diodes as a radiation source for plants. HortScience.

[4] Gupta S.D., Jatothu B. (2013). Fundamentals and applications of light-emitting diodes (LEDs) in in vitro plant growth and morphogenesis. Plant Biotechnol. Rep..

[5] Brown C.S., Schuerger A.C., Sager J.C. (1995). Growth and photomorphogenesis of pepper plants under red light-emitting diodes with supplemental blue or far-red lighting. J. Am. Soc. Hort. Sci..

[6] Yeh N., Chung J.-P. (2009). High-brightness LEDs—Energy efficient lighting sources and their potential in indoor plant cultivation. Renew. Sustain. Energy Rev..

[7] Li H., Xu Z., Tang C. (2010). Effect of light-emitting diodes on growth and morphogenesis of upland cotton (*Gossypium hirsutum* L.) plantlets in vitro. Plant Cell Tissue Organ Cult..

[8] Chen C.-C., Agrawal D.C., Lee M.-R., Lee R.-J., Kuo C.-L., Wu C.-R., Tsay H.-S., Chang H.-C. (2016). Influence of LED light spectra on in vitro somatic embryogenesis and LC–MS analysis of chlorogenic acid and rutin in *Peucedanum japonicum* Thunb.: A medicinal herb. Bot. Stud..

[9] Pawłowska B., Żupnik M., Szewczyk-Taranek B., Cioć M. (2018). Impact of LED light sources on morphogenesis and levels of photosynthetic pigments in *Gerbera jamesonii* grown in vitro. Hortic. Environ. Biotechnol..

[10] Kubica P., Szopa A., Prokopiuk B., Komsta Ł., Pawłowska B., Ekiert H. (2020). The influence of light quality on the production of bioactive metabolites–verbascoside, isoverbascoside and phenolic acids and the content of photosynthetic pigments in biomass of *Verbena officinalis* L. cultured in vitro. J. Photochem. Photobiol. B Biol..

[11] Tryon R. (1976). A revision of the genus *Cyathea*. Contrib. Gray Herb. Harvard Univ..

[12] Moran R.C., Klimas S., Carlsen M. (2003). Low-trunk epiphytic ferns on tree ferns versus angiosperms in Costa Rica. Biotropica.

[13] CITES (2021). Convention of International Trade in Endangered Species of Wild Fauna and Flora. https://cites.org/eng/app/appendices.php.

[14] Eleutério A.A., Pérez-Salicrup D. (2006). Management of tree ferns (*Cyathea* spp.) for handicraft production in Cuetzalan, Mexico. Econ. Bot..

[15] Liu Y., Wujisguleng W., Long C. (2012). Food uses of ferns in China: A review. Acta Soc. Bot. Pol..

[16] Goswami H.K., Sen K., Mukhopadhyay R. (2016). Pteridophytes: Evolutionary boon as medicinal plants. Plant Genet. Resour..

[17] Nath K., Talukdar A.D., Bhattacharya M.K., Bhowmik D., Chetri S., Choudhury D., Mitra A., Choudhury N.A. (2019). *Cyathea gigantea* (Cyatheaceae) as an antimicrobial agent against multidrug resistant organisms. BMC Complement. Altern..

[18] Janakiraman N., Johnson M. (2016). Larvicidal potential of *Cyathea* species against *Culex quinquefasciatus*. Pharm. Biomed. Res..

[19] Ida N., Iwasaki A., Teruya T., Suenaga K., Kato-Noguchi H. (2020). Tree fern *Cyathea lepifera* may survive by its phytotoxic property. Plants.

[20] Madhu Kiran P., Vijaya Raju A., Ganga Rao B. (2012). Investigation of hepatoprotective activity of *Cyathea gigantea* (Wall. ex. Hook.) leaves against paracetamol-induced hepatotoxicity in rats. Asian Pac. J. Trop. Biomed..

[21] Faizal A., Taufik I., Rachmani A.F., Prihartini Azar A.W. (2020). Short communication: Antioxidant and antibacterial properties of tree fern *Cyathea contaminans*. Biodiversitas.

[22] Hiendlmeyer R., Randi A.M. (2007). Response of spores and young gametophytes of *Cyathea delgadii* Sternb. (Cyatheaceae) and *Blechnum brasiliense* Desv. (Blechnaceae) to different light levels. Acta Bot. Bras..

[23] Rechenmacher C., Schmitt J.L., Droste A. (2010). Spore germination and gametophyte development of *Cyathea atrovirens* (Langsd. & Fisch.) Domin (Cyatheaceae) under different pH conditions. Braz. J. Biol..

[24] Reis Moura I., Simões-Costa M.C., Garcia J., Silva M.J., Duarte M.C. (2012). In vitro culture of tree fern spores from Cyatheaceae and Dicksoniaceae families. Acta Hortic..

[25] Das S., Dutta Choudhury M., Mazumder P.B. (2013). Research article in vitro propagation of *Cyathea gigantea* (wall ex. Hook)-A tree fern. Int. J. Recent Sci. Res..

[26] Marcon C., Silveira T., Schmitt J.L., Droste A. (2017). Abiotic environmental conditions for germination and development of gametophytes of *Cyathea phalerata* Mart. (Cyatheaceae). Acta Bot. Brasilica.

[27] Rybczyński J.J., Mikuła A., Fernández H. (2011). Tree ferns biotechnology: From spores to sporophytes. Working with Ferns: Issues and Applications.

[28] Bonomo M.C., Martínez O.G., Tanco M.E., Cardozo R., Avilés Z. (2013). Spores germination and gametophytes of *Alsophila odonelliana* (Cyatheaceae) in different sterile media. Pyton.

[29] Yu R., Zhang G., Li H., Cao H., Mo X., Gui M., Zhou X., Jiang Y., Li S., Wang J. (2017). In vitro propagation of the endangered tree fern *Cibotium barometz* through formation of green globular bodies. Plant Cell Tiss. Organ Cult..

[30] Mikuła A., Pożoga M., Tomiczak K., Rybczyński J.J. (2015). Somatic embryogenesis in ferns: A new experimental system. Plant Cell Rep..

[31] Parajuli J., Joshi S.D. (2014). In vitro study of effects of growth hormones on sporophyte development of *Cyathea spinulosa*. Int. J. Biodivers. Conserv..

[32] Shukla S.P., Khare P.B. (2014). In vitro conservation of some threatened and economically important ferns belonging to the Indian subcontinent. J. Bot..

[33] Mikuła A., Pożoga M., Grzyb M., Rybczyński J.J. (2015). An unique system of somatic embryogenesis in the tree fern *Cyathea delgadii* Sternb.: The importance of explant type, and physical and chemical factors. Plant Cell Tiss. Organ Cult..

[34] Grzyb M., Mikuła A. (2019). Explant type and stress treatment determine the uni-and multicellular origin of somatic embryos in the tree fern *Cyathea delgadii* Sternb. Plant Cell Tiss. Organ Cult..

[35] Grzyb M., Kalandyk A., Waligórski P., Mikuła A. (2017). The content of endogenous hormones and sugars in the process of early somatic embryogenesis in the tree fern *Cyathea delgadii* Sternb. Plant Cell Tiss. Organ Cult..

[36] Gauer Medeiros L., Marcon C., Silveira T., Schmitt J.L., Droste A. (2017). Looking for the conservation and sustainable use of *Cyathea corcovadensis* (Raddi) Domin (Cyatheaceae): The influence of environmental factors on gametophytes. Braz. J. Bot..

[37] Donaher D.J., Partanen C.R. (1971). The role of light in the interrelated processes of morphogenesis and photosynthesis in the fern gametophyte. Physiol. Plant..

[38] Wada M., Sei H. (1994). Phytochrome-mediated phototropism in *Adiantum cuneatum* young leaves. J. Plant Res..

[39] Sugiyama Y., Kadota A. (2011). Photosynthesis-dependent but neochrome1-independent light positioning of chloroplasts and nuclei in the fern *Adiantum capillus-veneris*. Plant Physiol..

[40] Doi M., Wada M., Shimazaki K.-I. (2006). The fern *Adiantum capillus-veneris* lacks stomatal responses to blue light. Plant Cell Physiol..

[41] Murashige T., Skoog F. (1962). A revised medium for rapid growth and bio assays with tobacco tissue cultures. Physiol. Plant..

[42] Lichtenthaler H.K., Buschmann C. (2001). Chlorophylls and carotenoids: Measurement and characterization by UV-VIS spectroscopy. Curr. Protoc. Food Anal. Chem..

[43] Khasanah N.M., Isnansetyo A., Nuringtyas T.R. (2017). Root anatomy and growth responses of soybean (Glycine max (L.) Merr.) ‘Wilis’ to NaCl stress. Proceedings of the 1st International Conference on Tropical Agriculture.

[44] Pietrzak W., Nowak R., Gawlik-Dziki U., Lemieszek M.K., Rzeski W. (2017). LC-ESI-MS/MS identification of biologically active phenolic compounds in mistletoe berry extracts from different host trees. Molecules.

[45] Olech M., Nowacka-Jechalke N., Masłyk M., Martyna A., Pietrzak W., Kubiński K., Załuski D., Nowak R. (2019). Polysaccharide-rich fractions from *Rosa rugosa* Thunb.-Composition and chemopreventive potential. Molecules.

[46] Guo J.-T., Lee H.-L., Chiang S.-H., Lin F.-I., Chang C.-Y. (2001). Antioxidant properties of the extracts from different parts of broccoli in Taiwan. J. Food Drug Anal..

[47] Szewczyk K., Bogucka-Kocka A., Vorobets N., Grzywa-Celińska A., Granica S. (2020). Phenolic composition of the leaves of *Pyrola rotundifolia* L. and their antioxidant and cytotoxic activity. Molecules.

[48] Deba F., Xuan T.D., Yasuda M., Tawata S. (2008). Chemical composition and antioxidant, antibacterial and antifungal activities of the essential oils from *Bidens pilosa* Linn. var. Radiata. Food Control.

[49] Hutchinson M.J., Senaratna T., Sahi S.V., Saxena P.K. (2000). Light mediates endogenous plant growth substances in thidiazuron-induced somatic embryogenesis in geranium hypocotyl cultures. J. Plant Biochem. Biotechnol..

[50] Gatica A.M., Arrieta G., Espinoza A.M. (2008). Direct somatic embryogenesis in *Coffea arabica* L. CVS. caturra and catuaí: Effect of triacontanol, light condition, and medium consistency. Agron. Costarricense.

[51] Park S.-Y., Yeung E.C., Paek K.-Y. (2010). Endoreduplication in *Phalaenopsis* is affected by light quality from light-emitting diodes during somatic embryogenesis. Plant Biotechnol. Rep..

[52] Lee S.-H., Kumar Tewari R., Hahn E.-J., Paek K.-Y. (2007). Photon flux density and light quality induce changes in growth, stomatal development, photosynthesis and transpiration of *Withania Somnifera* (L.) Dunal. plantlets. Plant Cell Tiss. Organ Cult..

[53] Aalifar M., Arab M., Aliniaeifard S., Dianati S., Mehrjerdi M.Z., Limpens E., Serek M. (2019). Embryogenesis efficiency and genetic stability of *Dianthus caryophyllus* embryos in response to different light spectra and plant growth regulators. Plant Cell Tiss. Organ Cult..

[54] De Castro K.M., Batista D.S., Fortini E.A., Silva T.D., Felipe S.H.S., Fernandes A.M., de Jesus Sousa R.M., de Queiroz Nascimento L.S., Campos V.R., Grazul R.M. (2019). Photoperiod modulates growth, morphoanatomy, and linalool content in *Lippia alba* L. (Verbenaceae) cultured in vitro. Plant Cell Tiss. Organ Cult..

[55] Macedo A.F., Leal-Costa M.V., Tavares E.S., Lage C.L.S., Esquibel M.A. (2011). The effect of light quality on leaf production and development of in vitro-cultured plants of *Alternanthera brasiliana* Kuntze. Environ. Exp. Bot..

[56] Cybularz-Urban T., Hanus-Fajerska E., Świderski A. (2007). Effect of light wavelength on in vitro organogenesis of a *Cattleya* hybrid. Acta Biol. Crac. Ser. Bot..

[57] Ryu J.H., Seo K.S., Choi G.L., Rha E.S., Lee S.C., Choi S.K., Kang S.-Y., Bae C.-H. (2012). Effects of LED Light illumination on germination, growth and anthocyanin content of dandelion (*Taraxacum officinale*). Korean J. Plant Res..

[58] Wu H.-C., Lin C.-C. (2012). Red light-emitting diode light irradiation improves root and leaf formation in difficult-to-propagate *Protea cynaroides* L. plantlets in vitro. HortScience.

[59] Lazzarini L.E.S., Bertolucci S.K.V., Pacheco F.V., dos Santos J., Silva S.T., de Carvalho A.A., Pinto J.E.B.P. (2018). Quality and intensity of light affect *Lippia gracilis* Schauer plant growth and volatile compounds in vitro. Plant Cell Tiss. Organ Cult..

[60] Tester M., Morris C. (1987). The penetration of light through soil. Plant Cell Environ..

[61] Silva-Navas J., Moreno-Risueno M.A., Manzano C., Pallero-Baena M., Navarro-Neila S., Téllez-Robledo B., Garcia-Mina J.M., Baigorri R., Gallego F.J., del Pozo J.C. (2015). D-Root: A system for cultivating plants with the roots in darkness or under different light conditions. Plant J..

[62] Díaz-Pérez J.C., Shackel K.A., Sutter E.G. (1995). Effects of in vitro-formed roots and acclimatization on water status and gas exchange of tissue-cultured apple shoots. J. Amer. Soc. Hort. Sci..

[63] Kircher S., Schopfer P. (2012). Photosynthetic sucrose acts as cotyledon-derived long-distance signal to control root growth during early seedling development in *Arabidopsis*. Proc. Natl. Acad. Sci. USA.

[64] Sassi M., Lu Y., Zhang Y., Wang J., Dhonukshe P., Blilou I., Dai M., Li J., Gong X., Jaillais Y. (2012). COP1 mediates the coordination of root and shoot growth by light through modulation of PIN1-and PIN2-dependent auxin transport in *Arabidopsis*. Development.

[65] Su N., Wu Q., Shen Z., Xia K., Cui J. (2014). Effects of light quality on the chloroplastic ultrastructure and photosynthetic characteristics of cucumber seedlings. Plant Growth Regul..

[66] Liu M., Xu Z., Yang Y., Yijie F. (2011). Effects of different spectral lights on *Oncidium* PLBs induction, proliferation, and plant regeneration. Plant Cell Tiss. Organ Cult..

[67] Silva S.T., Bertolucci S.K.V., da Cunha S.H.B., Lazzarini L.E.S., Tavares M.C., Pinto J.E.B.P. (2017). Effect of light and natural ventilation systems on the growth parameters and carvacrol content in the in vitro cultures of *Plectranthus amboinicus* (Lour.) Spreng. Plant Cell Tiss. Organ Cult..

[68] Wu H., Liu X.-G., Ji H., Chen L.-Q. (2010). Effects of light, macronutrients, and sucrose on germination and development of the endangered fern *Adiantum reniforme* var. sinense (Adiantaceae). Sci. Hortic..

[69] Suetsugu N., Wada M. (2003). Cryptogam blue-light photoreceptors. Curr. Opin. Plant Biol..

[70] Wada M., Kanegae T., Nozue K., Fukuda S. (1997). Cryptogam phytochromes. Plant Cell Environ..

[71] Grill R. (1987). Induction of two-dimensional growth by red and green light in the fern *Anemia phyllitidis* L. Sw. J. Plant Physiol..

[72] Sobota A.E., Partanen C.R. (1965). The growth and division of cells in relation to morphogenesis in fern gametophytes: I. Photomorphogenetic studies in *Pteridium aquilinum*. Can. J. Bot..

[73] Niranjan A.R.S., Singh I.P., Roy S.K. (1983). Effect of UV-irradiation on the growth and differentiation of the gametophyte in the fern *Cheilanthes rufa* D. Proc. Indian Natl..

[74] Randi A.M., Freitas M.C.A., Rodrigues A.C., Maraschin M., Torres M.A. (2014). Acclimation and photoprotection of young gametophytes of *Acrostichum danaeifolium* to UV-B stress. Photosynthetica.

[75] Fan X.X., Zang J., Xu Z.G., Guo S.R., Jiao X.L., Liu X.Y., Gao Y. (2013). Effects of different light quality on growth, chlorophyll concentration and chlorophyll biosynthesis precursors of non-heading Chinese cabbage (*Brassica campestris* L.). Acta Physiol. Plant..

[76] Hung C.D., Hong C.-H., Kim S.-K., Lee K.-H., Park J.-Y., Nam M.-W., Choi D.-H., Lee H.-I. (2016). LED light for in vitro and ex vitro efficient growth of economically important highbush blueberry (*Vaccinium corymbosum* L.). Acta Physiol. Plant..

[77] Ye S., Shao Q., Xu M., Li S., Wu M., Tan X., Su L. (2017). Effects of light quality on morphology, enzyme activities, and bioactive compound contents in *Anoectochilus roxburghii*. Front. Plant Sci..

[78] Cioć M., Szewczyk A., Żupnik M., Kalisz A., Pawłowska B. (2018). LED lighting affects plant growth, morphogenesis and phytochemical contents of *Myrtus communis* L. in vitro. Plant Cell Tiss. Organ Cult..

[79] Khayatnezhad M., Gholamin R., Jamaati-e-Somarin S., Zabihi-e-Mahmoodabad R. (2011). The leaf chlorophyll content and stress resistance relationship considering in corn cultivars (*Zea mays*). Adv. Environ. Biol..

[80] Cioć M., Pawłowska B. (2020). Leaf response to different light spectrum compositions during micropropagation of *Gerbera* axillary shoots. Agronomy.

[81] Liu X.Y., Guo S.R., Xu Z.G., Jiao X.L., Takafumi T. (2011). Regulation of chloroplast ultrastructure, cross-section anatomy of leaves, and morphology of stomata of cherry tomato by different light irradiations of light-emitting diodes. HortScience.

[82] Miao Y.-X., Wang X.-Z., Gao L.-H., Chen Q.-Y., Qu M. (2016). Blue light is more essential than red light for maintaining the activities of photosystem II and I and photosynthetic electron transport capacity in cucumber leaves. J. Integr. Agric..

[83] Simlat M., Ślęzak P., Moś M., Warchoł M., Skrzypek E., Ptak A. (2016). The effect of light quality on seed germination, seedling growth and selected biochemical properties of *Stevia rebaudiana* Bertoni. Sci. Hortic..

[84] Zheng L., Van Labeke M.-C. (2017). Long-term effects of red-and blue-light emitting diodes on leaf anatomy and photosynthetic efficiency of three ornamental pot plants. Plants. Front. Plant Sci..

[85] Oksman-Caldentey K.-M., Inzé D. (2004). Plant cell factories in the post-genomic era: New ways to produce designer secondary metabolites. Trends Plant Sci..

[86] Landi M., Zivcak M., Sytar O., Brestic M., Allakhverdiev S.I. (2020). Plasticity of photosynthetic processes and the accumulation of secondary metabolites in plants in response to monochromatic light environments: A review. BBA Bioenerg..

[87] Lu H., Tian Z., Cui Y., Liu Z., Ma X. (2020). Chlorogenic acid: A comprehensive review of the dietary sources, processing effects, bioavailability, beneficial properties, mechanisms of action, and future directions. Compr. Rev. Food Sci. Food Saf..

[88] Kapoor S., Raghuvanshi R., Bhardwaj P., Sood H., Saxena S., Chaurasia O.P. (2018). Influence of light quality on growth, secondary metabolites production and antioxidant activity in callus culture of *Rhodiola imbricata* Edgew. J. Photochem. Photobiol. B Biol..

[89] Yap E.S.P., Uthairatanakij A., Laohakunjit N., Jitareerat P., Vaswani A., Magana A.A., Morre J., Maier C.S. (2021). Plant growth and metabolic changes in ‘Super Hot’ chili fruit (*Capsicum annuum*) exposed to supplemental LED lights. Plant Sci..

[90] Wang P., Chen S., Gu M., Chen X., Chen X., Yang J., Zhao F., Ye N. (2020). Exploration of the effects of different blue LED light intensities on flavonoid and lipid metabolism in tea plants via transcriptomics and metabolomics. Int. J. Mol. Sci..

[91] Hiraoka A., Hasegawa M. (1975). Flavonoid glycosides from five *Cyathea* species. Bot. Mag..

[92] Sharma P., Jha A.B., Dubey R.S., Pessarakli M. (2012). Reactive oxygen species, oxidative damage, and antioxidative defense mechanism in plants under stressful conditions. J. Bot..

