# Supplementary material for: Enhancing In Vitro Production of the Tree Fern Cyathea delgadii and Modifying Secondary Metabolite Profiles by LED Lighting
Source: Cells. 2022 Jan 30;11(3):486. doi: 10.3390/cells11030486 (PMC8834616; doi:10.3390/cells11030486)
Supplement: Supplementary file 1 [file cells-11-00486-s001.zip › cells-1563281-supplementary.pdf]

Supplementary Table S1. The list of secondary metabolites analysed in the extracts from *Cyathea delgadii*, growing under various light conditions, but not detected or at concentration higher than the limit of detection but lower than the limit of quantification.

Compound	Retention time [min]	[M-H]- [*m/z*]	Fragment ions [*m/z*]	Colision energy [eV]	Light conditions					
					Dark	Fl	B	R	RB	Greenhouse
					Compound Content [μg/mg of dry weight]					
Phenolic acids										
Gallic acid	5.14	168.7	78.9<br>124.9	-36<br>-14	nd	nd	nd	BQL	nd	nd
4-Hydroxycinnamic acid (*p*-coumaric acid)	14.05	162.7	119<br>93	-14<br>-44	nd	nd	BQL	BQL	BQL	nd
Trans-ferulic acid	14.82	192.8	133.9<br>177.9	-16<br>-12	nd	nd	nd	nd	nd	nd
Cis-ferulic acid	15.25	192.8	133.9<br>177.9	-16<br>-12	nd	nd	nd	nd	nd	nd
Flavonoid aglycones										
Luteolin	17.80	284.7	132.9<br>150.9	-38<br>-26	nd	nd	nd	nd	nd	BQL
Eriodictyol	17.85	286.7	134.9<br>150.9	-32<br>-18	nd	BQL	nd	BQL	nd	BQL
Quercetin	17.90	300.7	150.9<br>178.8	-26<br>-20	nd	BQL	BQL	BQL	BQL	nd
Apigenin	18.65	268.8	117<br>106.8	-44<br>-34	BQL	BQL	BQL	BQL	BQL	BQL
Kaempferol	18.86	284.7	116.8<br>93	-46<br>-52	nd	BQL	BQL	BQL	BQL	nd
Sakuranetin	21.68	284.7	118.9<br>164.8	-34<br>-20	nd	BQL	BQL	BQL	BQL	nd
Flavonoid glycosides										
Kaempferol 3,7-dirhamnoside (Kaempferitrin)	12.17	576.8	284.8<br>430.9	-42<br>-30	BQL	BQL	BQL	BQL	BQL	nd
Luteolin 7-*O*-glucoside (Luteoloside)	12.89	446.8	284.8<br>132.9	-30<br>-78	nd	nd	nd	nd	nd	nd

Fl—control light: fluorescence Philips TL-D 36W/54 lamps; B—100% blue LED light (430 nm); R—100% red LED light (670 nm); RB—combination of red (70%) and blue (30%) LED light. Cultures were maintained under a 16/8 h light/dark.; n=6; nd—not detected; BQL—peak detected, concentration higher than the limit of detection but lower than the limit of quantification.
